# Performance and cardiac evaluation before and after a 3-week training camp for 400-meter sprinters – An observational, non-randomized study

**DOI:** 10.1371/journal.pone.0217856

**Published:** 2019-05-31

**Authors:** Michael Skalenius, C. Mikael Mattsson, Pia Dahlberg, Lennart Bergfeldt, Annica Ravn-Fischer

**Affiliations:** 1 Department of Molecular and Clinical Medicine/Cardiology, Sahlgrenska University Hospital, Gothenburg, Sweden; 2 Department of Physiology and Pharmacology, Karolinska Institutet, Stockholm, Sweden; 3 Silicon Valley Exercise Analytics (SVExA), Menlo Park, CA, United States of America; 4 Region Västra Götaland, Sahlgrenska University Hospital, Department of Cardiology, Gothenburg, Sweden; Victoria University, AUSTRALIA

## Abstract

**Objective:**

To study the performance and cardiovascular function after a 3-week training camp in athletes competing in an anaerobically dominant sport.

**Methods:**

Twenty-three competitive 400-m athletes were enrolled in this non-randomized study, 17 took part in a 3-week training camp in South-Africa (intervention), but one declined follow-up assessment, while 6 pursued in-door winter training in Sweden and served as controls. Electrocardiography, transthoracic echocardiography, blood test analyses, maximal exercise tolerance test, and a 300-m sprint test with lactate measurements ([La]peak) were performed before and after the training camp period.

**Results:**

At baseline, there were no clinically significant pathological findings in any measurements. The training period resulted in improved 300m-sprint performance [n = 16; running time 36.71 (1.39) vs. 35.98 (1.13) s; p<0.01] and higher peak lactate values. Despite 48% more training sessions than performed on home ground (n = 6), myocardial biomarkers decreased significantly (NT-pro BNP -38%; p<0.05, troponin T -16%; p<0.05). Furthermore, resting heart rate (-7%; p<0.01) and left ventricular systolic and diastolic volumes decreased -6% (p<0.01) and -10% (p<0.05), respectively.

**Conclusions:**

Intense physical activity at training camp improved the performance level, likely due to improved anaerobic capacity indicated by higher [La]peak. There were no clinically significant adverse cardiac changes after this period of predominantly anaerobic training.

## Introduction

Participation in sports and exercise is an important lifestyle element for many people in the industrialized part of the world and generally considered beneficial for overall health [1]. Furthermore, there is a strong public interest in competitive sports even at the highest level [2]. For many athletes, participation at competitive level involves strenuous exercise and training, often inducing high cardiovascular load. It is therefore not surprising that specific electrocardiographic (ECG) features have been found in athletes compared with non-athletes as well as altered cardiac dimensions on transthoracic echocardiography (TTE) [3]. Of concern is, however, a small but not negligible risk of sudden cardiac death (SCD) among competitive athletes and pre-participation evaluation and its components are topics of debate [4, 5].

Most cardiovascular studies comparing athletes and non-athletes have focused on participants in endurance sports involving predominantly the aerobic energy system. Furthermore, interventional studies are scarce but have been conducted on soccer players [6]. The Olympic and World Championship category for sprint running is the category of races from 100 m (60 m indoor) to 400 m. Races from 200 m to 400 m are traditionally denominated as “long sprint”. No previous work that we know of has studied the cardiac function in performers of competitive long sprint running, such as 400-m. Competitive 400-m running is characterized by a relatively short predominantly anaerobic exercise where the athlete simultaneously often reaches maximal or near-maximal heart rates (HR) [7, 8]. The training regime is therefore mainly targeting anaerobic effect and capacity, but also includes exercises designed to improve strength, power, agility and aerobic capacity.

Limited knowledge is available about the cardiovascular function and effects of training in 400-m runners. During the winter, and in preparation for the indoor competitions season, Swedish athletes in track and field have an opportunity to participate in an annual training camp in South-Africa from end of December to early January. This provides an occasion to study competitive 400-m runners before and after a well-defined training camp. Our assumption was that performance would improve, as was the athletes’ goal. The aims of the comprehensive cardiovascular exam were to rule out any condition which would make intense training contra-indicated and to study any cardiovascular effects of the intense training period using standard medical procedures. These aims were achieved and an explanation for the improvement in sprint performance was offered.

## Materials and methods

### Participants

We enrolled 23 men of European descent. Their mean personal best on 400m was 49.82 (1.44) s and all competed on the Swedish national elite level. Exclusion criteria were specializing in 400m running less than 1 year, performance level below Swedish national elite, and residing too far from Gothenburg where the study took place.

Recruitment was accomplished through contact with local sprint coaches. Prior to the study, potential participants were informed about the procedure, possible gains, hazards and side effects and had an opportunity for questioning. They were asked to contact the investigator (MS) when interested in further information or participation. This study was approved by the regional ethics committee in Gothenburg and performed in accordance with the Declaration of Helsinki; written informed consent was obtained from all participants.

At the planning stage of this observational, non-randomized study, it was not known how many participants would travel to South Africa. Seventeen athletes went to the training camp, while 6 athletes did not for various personal reasons, but pursued their regular in-door training at home. They were at the same competitive level and with the same training background and regime and were therefore assigned to serve as controls regarding the change of climate and to minimize any bias in interpreting some of the observer dependent results.

### Study protocol

The baseline tests were performed 8–14 days prior to and the follow-up tests within 14 days after the training camp in South Africa and between December 2014—February 2015. All tests, except the long sprint, were performed in a hospital setting by cardiologists and specially trained nurses and technicians. The participants were instructed to consume a light meal before the tests and urged not to perform any heavy exercise the day before. General health was assessed at the first visit. Medical history was obtained, and a physical examination was performed by a cardiologist. Two questionnaires were constructed to evaluate any risk for SCD. One questionnaire was derived from the Swedish Sports Federation (*Riksidrottsförbundet*). The second was based on combining the first questionnaire with recommendations from the *American Heart Association* [9]. Both questionnaires were part of the medical history evaluation.

TTE and venous blood sampling were performed first and, at the end of the hospital visit, a maximal exercise tolerance test (ETT). The ETT was preceded by vectorcardiography (VCG; for a sub-study), and electrocardiography (ECG). The baseline long sprint test (300-m) including blood lactate measurements was conducted on the in-door training ground on a separate occasion, while the test after the training camp was performed either outdoors in South-Africa or indoors in Sweden.

All participants were also instructed to keep a training diary during a ~12-week period, from approximately 5 weeks before to 3 weeks after the training camp.

TTE was performed by an experienced technician on a GE Healthcare Vivid E9 machine using the M5S-D probe with the participant in the left lateral supine position. Long- and short- axis as well as apical and subcostal views were used following a predefined hospital routine protocol. Recordings were interpreted by a cardiologist, who was not informed whether the participant had been at the training camp or not. Reference values from sex, age and body surface area-matched populations were used for comparisons [10].

Laboratory blood tests included haemoglobin (Hb), haematocrit (Hct) and myocardial biomarkers (NT-proBNP and troponin T). As a part of the general health assessment C-reactive protein (CRP), white blood cell count (WBC), platelet count, sodium (Na), potassium (K), creatinine, alanine aminotransferase (ALAT), aspartate aminotransferase (ASAT), alkaline phosphatase (ALP) and bilirubin were also included. Changes in plasma volume (PV) were calculated according to the formula of Dill and Costill based upon changes in Hb and Hct (ΔHb and ΔHct) [11]. All analyses were performed at the laboratory of the Sahlgrenska University Hospital according to standard routines.

VCG was recorded using an orthogonal Frank-lead system (X,Y,Z) modified for the supine position for a sub study [12]. The recordings were obtained with a CoroNet II system (Ortivus AB, Danderyd, Sweden) during 8 minutes at supine rest with eyes closed immediately before the standard 12-lead ECG followed by the ETT. In the present report, only HR and conduction intervals were used to represent baseline values and derived automatically from a 3-dimensional QRST complex.

A standard 12-lead ECG was recorded at 50mm/s, on a Cardiolex Lexor *D* with the software EC sense. HR and conduction intervals were taken from the automatic measurements (and compared with those from the VCG; see Results and Discussion). The rest of the interpretation was performed by two of the authors jointly (MS, LB). The morphologic analysis was based on the “International Recommendations for Electrocardiographic Interpretation in Athletes” and amplitude criteria for left ventricular hypertrophy according to Sokolow-Lyon were applied with a partition value of 38 mV [13, 14].

A maximal ETT was performed using a stationary bicycle ergometer. An incremental protocol was chosen to achieve a work duration between 6 and 10 minutes until exhaustion [15]. Participants weighing >70 kg started from 90 W while those weighing <70 kg started from 70 W. The work rate increments were 30 W per minute. Blood pressure was measured intermittently with cuff and a Doppler ultrasound probe over the radial artery. Exertion and dyspnea were estimated using Borg Rating of Perceived Exertion (RPE) 10-scale [16]. Reference values for the expected work rate and maximum HR were obtained from age, sex, height and weight-matched populations [17, 18].

Sprint tests and lactate measurements were based on previous studies and a discussion with participants and their coaches. It was decided that a 300-m sprint at maximum speed would be the most suitable long-sprint performance test [19]. The degree of exertion and dyspnoea was estimated using the Borg RPE 10-scale [16]. Running time was clocked manually by two experienced coaches [mean difference was 0.05 (0.04) s]. All participants performed the baseline tests on indoor venues, while some athletes (n = 8) performed the follow-up test at the end of the training camp at an outdoor arena in South Africa. Because race times differ between outdoor and indoor venues, the outdoor times were corrected by extrapolation from a study on running 200 m; the outdoor-times were multiplied by 1.020 [20].

Blood lactate levels [La] were measured after the run aiming at defining the peak level and the lactate removal rate. Capillary blood was analysed with a portable lactate measurement device (Lactate Scout; EKF Diagnostics, Cardiff, United Kingdom, coefficient of variation for repeated samples is 3%). Samples were taken at supine rest at the 1^st^, 8^th^, 15^th^ and 22^nd^ minute after the run. Lactate clearance rate was determined by the linear equation: *k* = Δ*y*/Δ*x* = [La^−^]_b_ at 22 minutes—[La^−^]_b_ at 15 minutes/7.

### Intervention

The training period in South Africa lasted 22.0 (1.6) days, the first 10 days at 1350 m altitude and the remainder at sea level. The training included aerobic exercises, speed endurance, short sprint, jumping, strength, core and agility exercises. The schedule was not designed with the intention to increase the quantity of speed and strength training. Instead, the training camp was part of the preparation for the indoor competition season (which started shortly after the training camp) and therefore involved elements of tapering (see Supporting Information S1 Fig and S2 Fig).

### Statistics

Statistical analyses were performed using the non-parametric Wilcoxon signed rank-test for within-group comparisons because of the limited number of participants. No between-group comparisons were made because there was no randomization involved. Mean (SD) was used for descriptive purposes and 95% confidence intervals (CI) when found appropriate. Analyses were performed using *IBM SPSS Statistics 25*. P-values <0.05 were considered statistically significant.

## Results

Baseline data for all 23 participants are presented in Tables 1 and 2. Twenty-two athletes participated on both occasions; there was one drop-out among the 17 participating in the training camp. Data before and after the training camp for 16 participants are therefore shown in Table 3.

**Table 1:** Baseline characteristics for all 23 participants. Data presented as mean (SD).

Age [yr]	20.9 (2.3)
Body weight [kg]	75 (6)
Height [m]	1.84 (0.06)
Body mass index [kg·m^-2^]	22.0 (1.4)
Mean arterial pressure [mmHg]	88 (6)
**Blood analyses**	
NT-proBNP [ng·L^-1^]	35 (18)
Troponin T [ng·L^-1^]	8 (3)
C-reactive protein [mg·L^-1^] (4)‡	2 (2)
White blood cell count [x10*9·L^-1^]	6 (2)
Hemoglobin [g·dL^-1^]	150 (8)
Hematocrit [L·L^-1^]	0.44 (0.02)
Platelet count [x10*9·L^-1^]	219 (41)
Sodium [mmol·L^-1^]	140 (2)
Potassium [mmol·L^-1^]	4.4 (0.2)
Creatinine [μmol·L^-1^]	95 (12)
Alanine aminotransferase [μkat·L^-1^]	0.52 (0.18)
Aspartate aminotransferase [μkat·L^-1^]	0.63 (0.24)
Alkaline phosphatase [μkat·L^-1^]	1.3 (0.5)
Bilirubin [μmol·L^-1^]	11 (4)
**Vectorcardiography**	
Heart rate [bpm]	54 (9)
PR [ms]	157 (18)
QRS [ms]	102 (10)
QT [ms]	421 (25)
QTc Hodges [ms]	416 (19)
**Electrocardiography**	
Heart rate [bpm]	59 (10)
PR [ms]	153 (17)
QRS [ms]	96 (9)
QT [ms]	400 (23)
QTc Hodges [ms]	399 (17)
**Exercise tolerance test**	
Highest observed heart rate [bpm]	186 (8)
% of predicted maximal heart rate	94 (4)
Wmax [load reached]	365 (36)
Wmax [% of predicted] according to (17)	115 (11)
Wmax [% of predicted] according to (18)	157 (15)
**Transthoracic echocardiogram**	
EF—Simpson [%] (1)‡	57 (5)
LV EDV [ml·m^-2^]†	65 (8)
LV ESV [ml·m^-2^]†	27 (4)
LV septal thickness [cm]	0.86 (0.08)
LV posterior wall thickness [cm]	0.90 (0.10)
LV mass index [g·m^-2^]†	88 (13)
E/A ratio	1.97 (0.31)

‡Missing data

†normalized to BSA (Body Surface Area).

NT-proBNP, N-terminal probrain natriuretic peptide; RPE 10-scale, Rating of Perceived Exertion (at test termination); EF, ejection fraction; LV, left ventricular; EDV, end-diastolic; ESV, end-systolic volume.

**Table 2:** Number and proportions with 95% confidence intervals (CI) of common and uncommon ECG alterations at baseline according to recommendations on ECG interpretations in athletes (Sharma et al, 2017 [14]); n = 23.

Abnormality	N (%; 95% CI)
**Common**	
Sinus bradycardia	5 (22%; 8–44)
Sinus arrhythmia	21 (91%; 73–98)
Incomplete right bundle branch block	3 (13%; 3–35)
Early repolarization	18 (78%; 56–92)
Isolated QRS voltage criteria for left ventricular hypertrophy	14 (61%: 39–80)
**Uncommon**	
Left atrial enlargement	1 (4%; 0.2–24)
Right-axis deviation	1 (4%; 0.2–24)

**Table 3:** Comparison of data at baseline vs. after intervention in 16 participants with 6 controls for reference. Data presented as mean (SD). Within-group comparisons using Wilcoxon’s sign rank test.

	Intervention (n = 16)		Control (n = 6)	
	Baseline	Follow-up	Baseline	Follow-up
**Blood sample analyses**				
Haemoglobin [g·dL^-1^]	150 (9)	151 (9)	151 (7)	149 (5)
Haematocrit [L·L^-1^]	0.43 (0.03)	0.46 (0.02)**	0.44 (0.02)	0.45 (0.02)
Sodium [mmol·L^-1^]	140 (2)	141 (2)	140 (1)	140 (2)
Potassium [mmol·L^-1^]	4.4 (0.2)	4.5 (0.2) (2)‡	4.4 (0.2)	4.7 (0.4)
Creatinine [μmol·L^-1^]	96 (14)	102 (14)*	94 (5)	100 (10)
NT-proBNP [ng·L^-1^]	35 (20)	22 (17)*	35 (14)	29 (20)
Troponin T [ng·L^-1^]	8 (4)	7 (2)* (1)‡	8 (2)	6 (1)*
**Vectorcardiography**				
Heart rate [bpm]	54 (7)	50 (6)**	51 (10)	49 (5)
PR [ms]	157 (20)	153 (19)	159 (15)	162 (12)
QRS [ms]	101 (11)	100 (10)	104 (8)	100 (10)
QT [ms]	419 (21)	424 (25)	433 (33)	434 (8)
QTc Hodges [ms]	414 (20)	409 (19)	420 (18)	418 (13)
**Electrocardiography**				
Heart rate [bpm]	59 (9)	58 (6)	57 (13)	58 (12)
PR [ms]	151 (17)	149 (12)	162 (12)	155 (14)
QRS [ms]	94 (9)	94 (9)	100 (8)	98 (10)
QT [ms]	395 (18)	396 (24)	416 (30)	408 (35)
QTc Hodges [ms]	393 (16)	393 (19)	411 (15)	405 (20)
**Exercise tolerance test**				
Highest observed heart rate [bpm]	186 (8)	185 (8)	187 (7)	186 (2) (1)‡
Wmax [load reached]	358 (35)	352 (37)	371 (35)	364 (20) (1)‡
**Transthoracic echocardiography**				
EF—Simpson [%]	59 (5)	60 (7)	60 (5) (1)‡	56 (3)
LV EDV [ml·m^-2^]†	66 (8)	62 (9)**	61 (6)	58 (4)
LV ESV [ml·m^-2^]†	27 (4)	25 (6)*	26 (4)	25 (1)
LV mass Index [g·m^-2^]†	89 (15)	86 (15)	87 (5)	87 (9)
E/A ratio	2.01 (0.35)	2.00 (0.43)	1.85 (0.19)	1.83 (0.28)
**Sprint tests 300 m**				
Race time [s]	36.71 (1.39) (3)‡	35.98 (1.13)** (2)‡	36.49 (0.71) (1)‡	36.34 (1.01) (1)‡
Exertion [Borg RPE]	7.2 (1.5) (3)‡	7.4 (1.6) (2)‡	7.0 (1.4) (1)‡	6.5 (1.3) (1)‡
Dyspnea [Borg RPE]	5.5 (1.6) (3)‡	5.8 (1.4) (2)‡	6.0 (1.6) (1)‡	5.5 (0.6) (1)‡

*p<0.05

**p<0.01.

‡Missing data.

†normalized to BSA (Body Surface Area).

NT-proBNP, N-terminal probrain natriuretic peptide; EF, ejection fraction; LV, left ventricular; EDV, end-diastolic volume; ESV, end-systolic volume; RPE, Rating of Perceived Exertion.

### Medical history and questionnaires

The overall health state of the participants was good. Many had experienced dizziness associated with exercise and a few had experienced chest pain/discomfort, excessive dyspnoea/fatigue, and near-syncope/an unusual feeling of faint; all these symptoms were related to strenuous exercise and were by the participants viewed as part of their sport.

### Echocardiography

At baseline three participants had an enlarged left ventricle (two also had amplitude criteria for LVH on ECG) and one had an enlarged left atrium. There was high E/A ratios, but not remarkably high LV mass, in comparison with our reference values [10]. Left ventricular volumes were significantly smaller by 6–10% at follow-up; Table 3.

### Blood tests

At baseline most blood analyses were within the hospital reference values, except mildly elevated creatinine (4 participants), troponin T (3 participants), ASAT (3 participants), potassium (2 participants) and ALP (2 participants); Table 1. At follow-up, haematocrit [4.6%; p<0.01] and creatinine [5.7%; p<0.05] had increased, while NT-proBNP [-37.8%; p<0.05] and troponin T [-15.8 and -25.5% in both groups; p<0.05 for both] had decreased; Table 3. Plasma volume decreased significantly in the intervention group but not in the control group [-4.5 (8.0) %; p<0.05 and -0.6 (7.3) % (non-significant)].

### Vectorcardiography and electrocardiography

HR and conduction intervals at the VCG and ECG recordings at baseline are shown in Table 1. For athletes”common and uncommon ECG alterations” are summarized in Table 2 [14]. Sinus arrhythmia, early repolarization) (six had J-waves) and/or voltage criteria for ventricular hypertrophy were observed in all 23. After intervention, HR was significantly lower [-7.4%; p<0.01]; Table 3.

### Exercise test

The ETT test was in general terminated due to leg fatigue. One participant developed signs of bronchial obstruction at the highest work loade. The HR and blood pressure reactions were normal. There were no significant differences in maximal HR or maximal workload between baseline and follow-up; Table 3.

### Sprint-tests and lactate measurement

**Fig 1:**
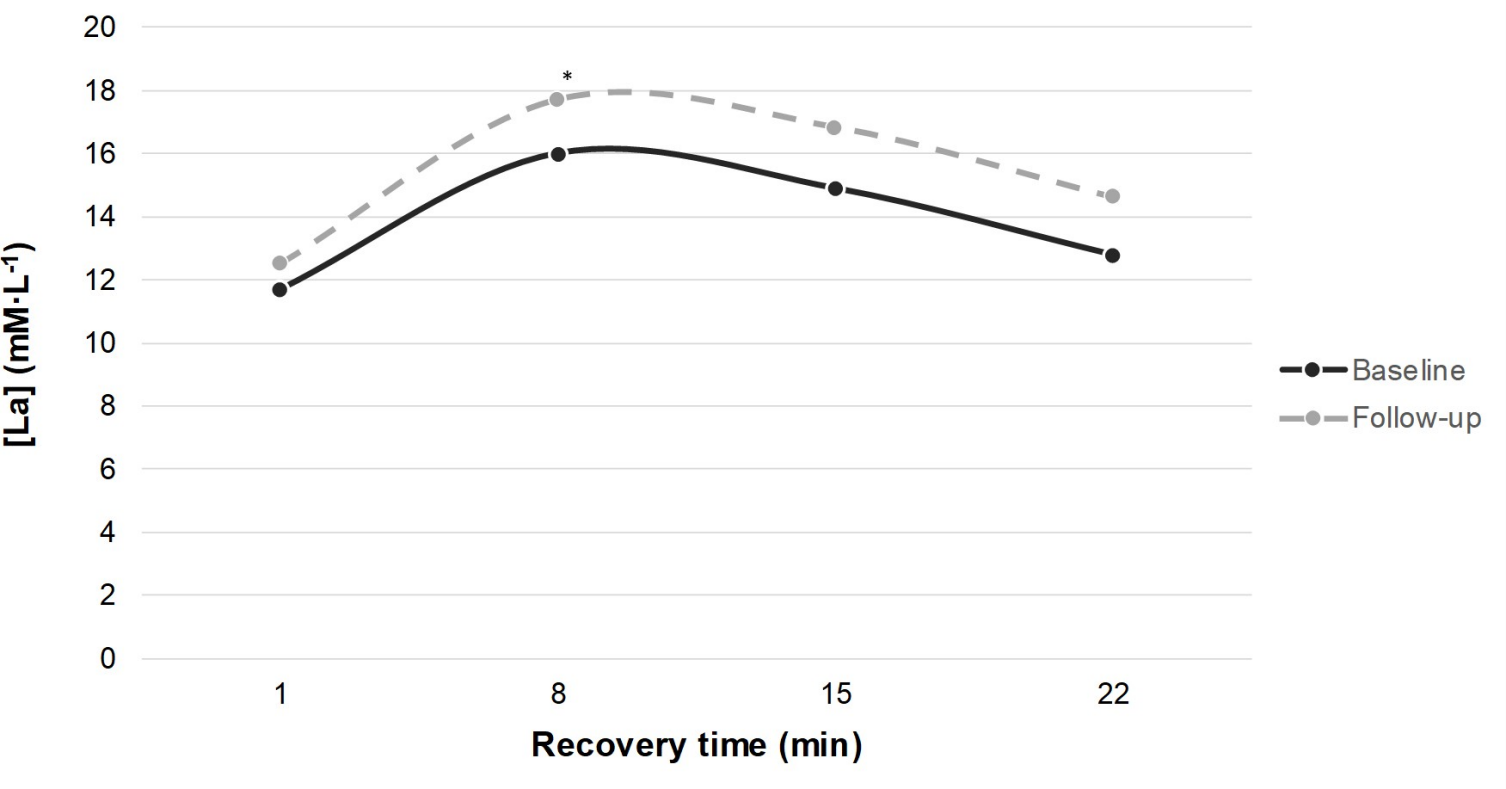
Blood lactate samples taken at supine rest at the 1^st^, 8^th^, 15^th^ and 22^nd^ minute after the 300 m run. Both races performed indoors, at 17–18°C. [La], blood lactate level. Baseline = solid line: Mean (SD) [La]peak: 16.2 (1.7) mM·L^-1^, Lactate removal rate: 0.310 (0.170) mM·L^-1^·min^-1^. Follow-up = dashed line: [La]peak: 17.8 (2.0) mM·L^-1^, Lactate removal rate: 0.316 (0.154) mM·L^-1^·min^-1^. *p<0.05. n = 9.

**Fig 2:**
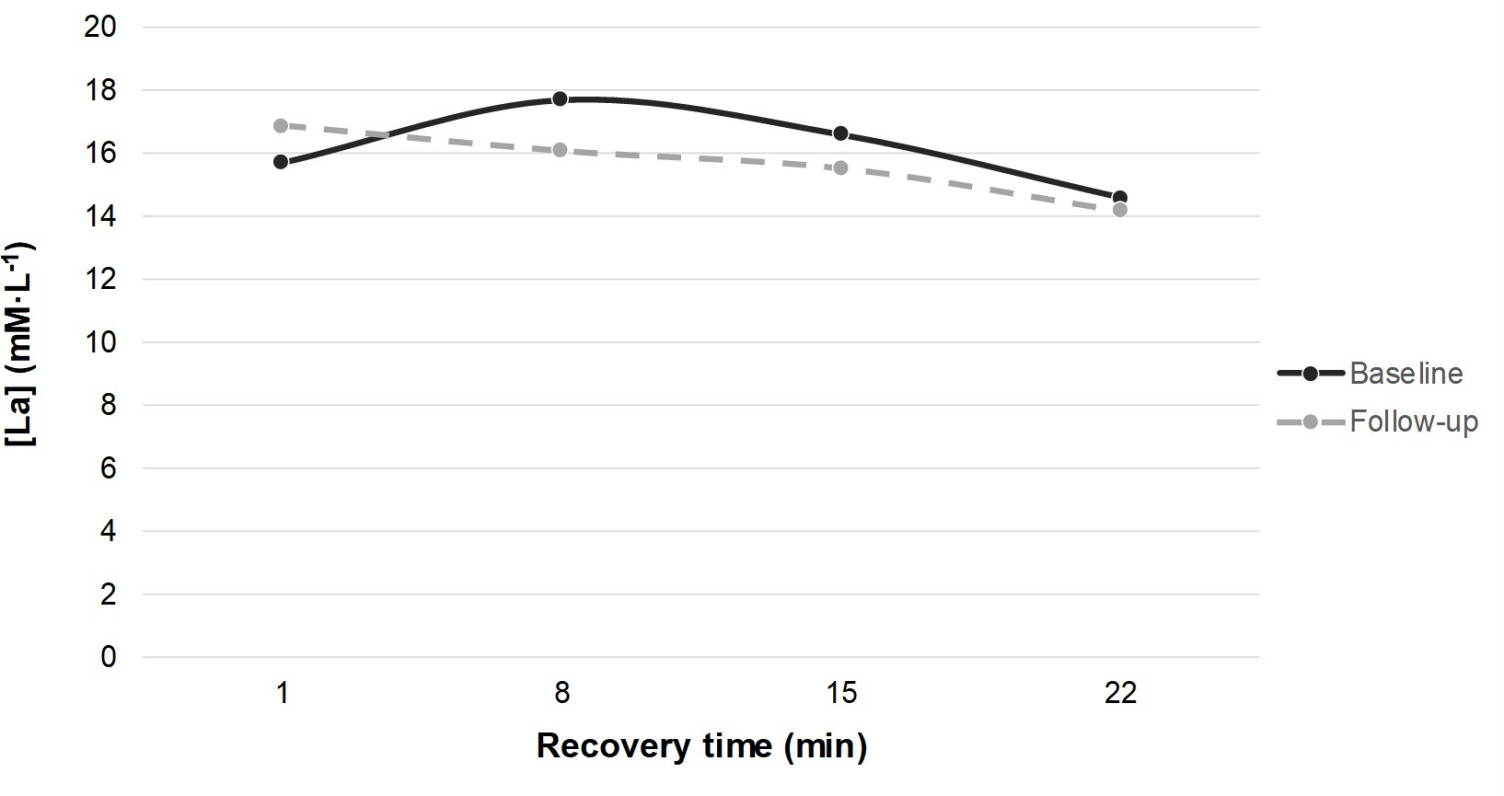
Blood lactate samples taken at supine rest at the 1^st^, 8^th^, 15^th^ and 22^nd^ minute after the 300 m run. Baseline race performed indoors, at 17–18°C. Follow-up race performed outdoors, at 30–31°C. [La], blood lactate level. Baseline = solid line: Mean (SD) [La]peak: 18.0 (2.9) mM L^-1^, Lactate removal rate: 0.284 (0.204) mM·L^-1^·min^-1^. Follow-up = dashed line: [La]peak: 17.0 (0.5) mM L^-1^, Lactate removal rate: 0.192 (0.121) mM·L^-1^·min^-1^. n = 8.

Five participants could not perform both races due to injuries, while nine of the remaining 17 performed both tests in-doors and eight performed the first test in-doors and the second out-doors in South Africa. Those who went to the training camp improved their race time by 0.73 (0.70) s (n = 14; p<0.01) without any significant difference in exertion or dyspnoea, while there was no improvement in the 4 who stayed home and performed both tests; Table 3. Results from the lactate analysis are presented in Figs 1 and 2.

There was no significant difference in the lactate analysis in either of the groups for all tests analysed together. However, when the outdoor times were excluded a significant difference was observed (a post hoc analysis); Fig 1. Comparing only in-door races, [La]peak increased by 10.1% at follow-up (n = 9; p<0.01), but there were no statistically significant changes in lactate removal rates. At another post hoc analysis, there were differences in the shape of the lactate curves; at follow-up, the lactate curves differed between indoor and outdoor tests; Fig 2. Seven of eight who ran their second 300-m race in South Africa had an estimated time point for [La]peak 7 minutes earlier compared with baseline (no difference in one participant); Fig 2. In contrast, among the nine athletes who ran both races in-doors, only two had their estimated [La]peak time-point earlier (7 minutes), two of them 7 minutes later, and in five athletes the points were unchanged; Fig 1.

## Discussion

We performed a thorough cardiovascular evaluation before and within 14 days after a 3-weeks training camp in South-Africa in 16 male competitive 400-m runners and a 300-m sprint test with lactate analyses before and after the training period. A “control group” of 6 runners of equal performance level underwent the same protocol, except the training camp. The performance of the sprint test improved after the camp and presumably due to improved lactate tolerance. There were no detectable cardiovascular adverse effects of the intensified training period.

The improvement in sprint time might seem paradoxical since training camps usually include higher training loads than normal training (in this case 48% more training sessions than on home ground), inducing more fatigue and consequently decreased performance. However, this specific training camp was scheduled in the taper period before the indoor competition season. Furthermore, most of these athletes were not professional and the training camp meant that they had no other obligations than training, eating prepared meals and more/better sleep opportunities. Increased sleep during regular training has been shown to significantly improve sprinting performance by 4.3% [21]. We therefore assume that our participants could tolerate and adapt to higher training loads than normal [22].

Improvement in long sprint is associated with higher maximal lactate levels [23]. A relationship with an augmentation of the anaerobic capacity is assumed for which the increase in measured [La]peak can serve as a proxy. In our study one explanation for the difference in time points for measured [La]peak between baseline and follow-up might be the difference in environmental temperature at the sprint test. We hypothesize that the efflux of lactate from muscle to blood occurs at a higher rate in a warm environment, because of cardiovascular compensation mechanisms (most likely altered peripheral circulation). Due to the unchanged lactate removal rates, increased activity of lactate degrading enzymes (lactate dehydrogenase) is unlikely an explanation. Changes in plasma volume might, however, affect the results.

We performed a thorough “pre-participation screening. Although most runners reported symptoms that would have been considered warning symptoms in relation to SCD risk, the comprehensive cardiovascular evaluation revealed no clinically significant abnormalities.

Several studies have reported an increase in NT-proBNP and troponin T levels after participation in endurance activities [24, 25]. We did not study the release of biomarkers in direct relation to exercise. NT-pro-BNP is excreted from the ventricle because of increased wall tension and our observations are in line with those of the TTE examination. Furthermore, Hct is a marker for hydration status, and it can be altered by a stay at a high altitude and exercise per se as well as by heat acclimatization [26, 27]. Also, the long flight (~13 hours) could have had an impact on the plasma volume. Consequently, and since Hb was stable, the increase in Hct was most likely due to the decreased plasma volume, which also could explain the increase in creatinine levels and contribute to reduced LV volumes at follow-up. TTE also showed markedly lower LV mass than in different types of male athletes and was not especially high compared to our reference values [3, 10]. The high E/A ratios (compared to reference values) were presumably due to a very good ventricular compliance in these young athlete’s hearts corroborating the results of a previous study [6].

The VCG recordings were performed for a sub-study and preceded the ECG and ETT. Our participants appeared more relaxed during the VCG recording, as judged from the lower resting HR compared with the subsequent ECG recording where some anticipation effects prior to the ETT seemed to occur. Baseline HR and conduction intervals were therefore obtained from the VCG recordings. All participants had ECG-alterations commonly found in athletes and assumed to be training-related [14]. A markedly higher proportion of them had LVH voltage criteria compared to results of a study performed on 1000 junior athletes using a lower separation criterion for LVH, [13, 28]. TTE did, however, not reveal any pathological hypertrophy among our participants, but their low BMI and slim constitution might contribute to high QRS amplitudes. Also, a relatively high proportion of our athletes had signs of early repolarization, although the prevalence of J-waves seems to be within the normal range of the athlete population [29, 30].

A major limitation of the interventional part of this study is its non-randomized design. All participants’ wish and ambition to take part in the training camp was, however, clear from the very beginning. Furthermore, the major goal was to evaluate any detrimental cardiovascular effect of an intense training period and the more participants the better power to detect any differences. The 6 participants training on home ground offered, however, an opportunity to minimize any observational bias in the TTE assessment, where some subjectivity is an inherent feature. It was performed without knowledge about the participant’s training location.

## Conclusions

The performance of a 300-m sprint test improved after three weeks of training in a warm environment and was presumably due to improved lactate tolerance. There were no detectable cardiovascular adverse effects of the intensified training period. On the contrary, we observed reduced LV volumes despite lower resting HR and decreases in plasma levels of myocardial biomarkers (NT-pro-BNP and troponin T) after this predominantly anaerobic type of training. Our observations extend the present knowledge of cardiovascular physiology in this subgroup of competitive athletes focusing on long-sprint performance.

### Practical implications

- Our observations suggest that cardiac alteration common in endurance athletes may also be found in predominantly anaerobically trained athletes, but without any pathological connotations.
- Even after a training camp including ~50% more training session than the athletes’ usual schedule no cardiovascular clinically adverse effects were detected.
- Our findings indicate that this type of training camp can be safely conducted from a cardiovascular perspective and can lead to improvements in long-sprint performance.

## Supporting information

S1 Fig Training types during one (average) week, the training camp presented as mean (SD), n = 17. (TIF)

S2 Fig Training types home ground (one week) presented as mean (SD), n = 6. (TIF)

S1 Dataset (XLSX)
