# Supplementary figures and images for: Performance and cardiac evaluation before and after a 3-week training camp for 400-meter sprinters – An observational, non-randomized study
Source: PLoS One. 2019 May 31;14(5):e0217856. doi: 10.1371/journal.pone.0217856 (PMC6544373; doi:10.1371/journal.pone.0217856)

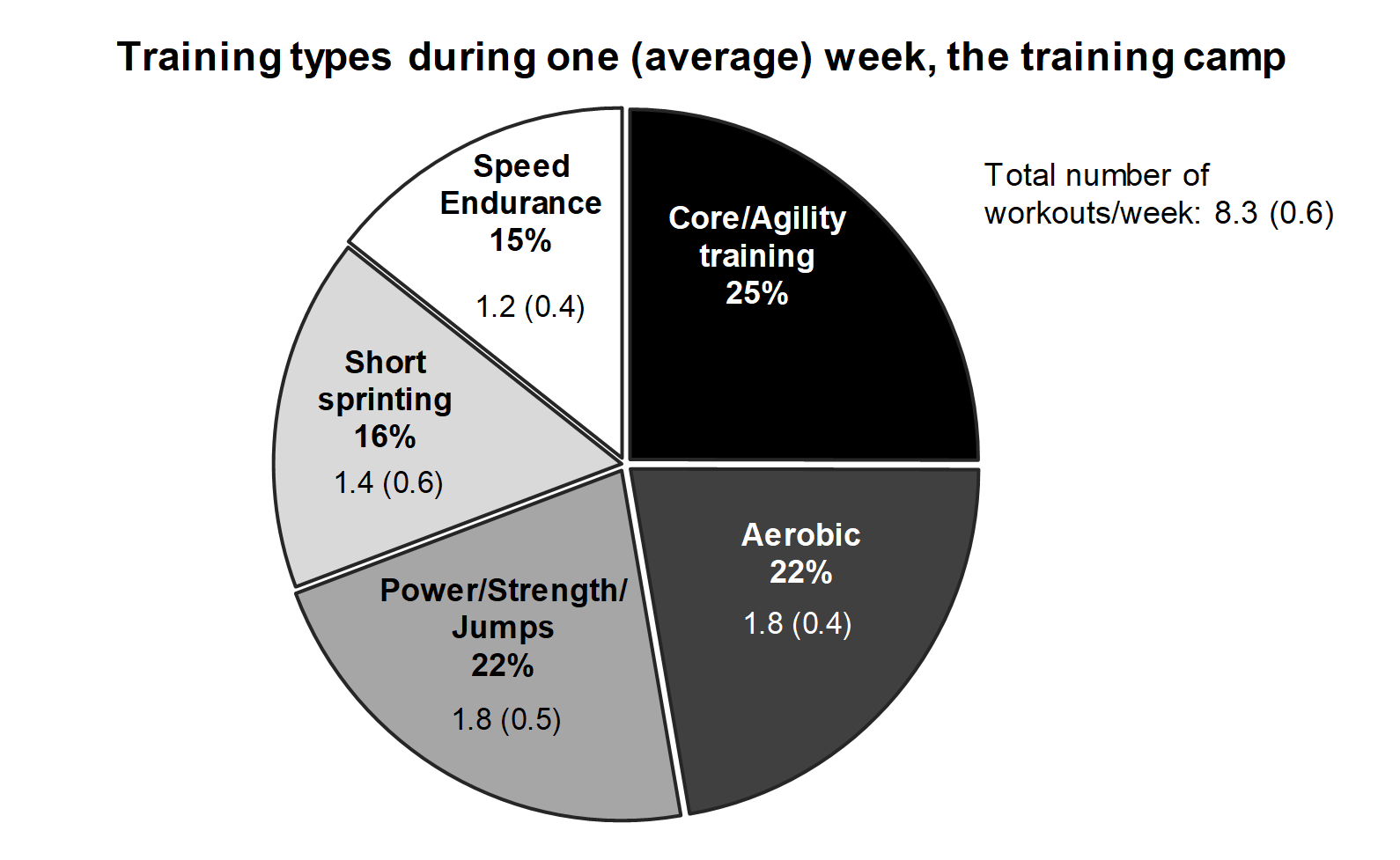

Supplement: S1 Fig — (TIF) [file pone.0217856.s001.tif]

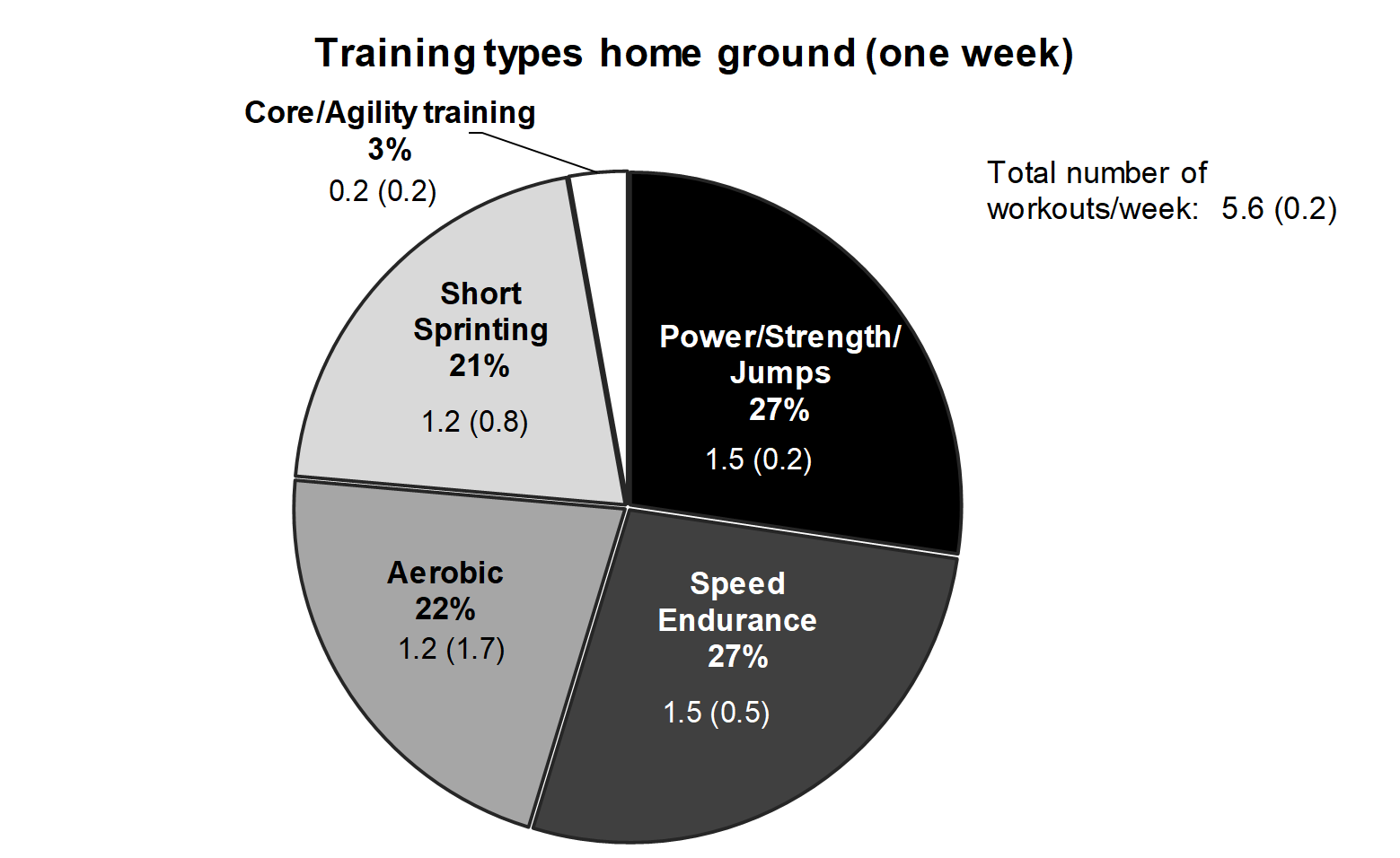

Supplement: S2 Fig — (TIF) [file pone.0217856.s002.tif]
